# Effect of Wear Conditions, Parameters and Sliding Motions on Tribological Characteristics of Basalt and Glass Fibre Reinforced Epoxy Composites

**DOI:** 10.3390/ma14030701

**Published:** 2021-02-02

**Authors:** Anis Adilah Abu Talib, Aidah Jumahat, Mohammad Jawaid, Napisah Sapiai, Alcides Lopes Leao

**Affiliations:** 1 Faculty of Mechanical Engineering, Universiti Teknologi MARA (UiTM), Shah Alam 40450, Malaysia; anisadilah86@gmail.com (A.A.A.T.); napisah@uitm.edu.my (N.S.); 2 Institute for Infrastructure Engineering Sustainable and Management (IIESM), Universiti Teknologi MARA, Shah Alam 40450, Malaysia; 3 Department of Biocomposite Technology, Institute of Tropical Forestry and Forest Products, Universiti Putra Malaysia, Selangor 43400, Malaysia; 4 Department of Natural Resources, College of Agricultural Sciences, São Paulo State University (UNESP), Botucatu 18610-307, Brazil; alcides.leao@unesp.br

**Keywords:** glass fibre, basalt fibre, polymer composites, adhesive wear, abrasive wear, erosive wear, reciprocating adhesive wear

## Abstract

Basalt fibre is a promising mineral fibre that has high potential to replace synthetic based glass fibre in today’s stringent environmental concern. In this study, friction and wear characteristics of glass and basalt fibres reinforced epoxy composites were studied and comparatively evaluated at two test stages. The first stage was conducted at fixed load, speed and distance under three different conditions; adhesive, abrasive and erosive wear, wherein each composite specimens slide against steel, silicon carbide, and sand mixtures, respectively. The second stage was conducted involving different types of adhesive sliding motions against steel counterpart; unidirectional and reciprocating motion, with the former varied at pressure—velocity (PV) factor; 0.23 MPa·m/s and 0.93 MPa·m/s, while the latter varied at counterpart’s configuration; ball-on-flat (B-O-F) and cylinder-on-flat (C-O-F). It was found that friction and wear properties of composites are highly dependent on test conditions. Under 10 km test run, Basalt fibre reinforced polymer (BFRP) composite has better wear resistance against erosive sand compared to Glass fibre reinforced polymer (GFRP) composite. In second stage, BFRP composite showed better wear performance than GFRP composite under high PV of unidirectional sliding test and under B-O-F configuration of reciprocating sliding test. BFRP composite also exhibited better friction properties than GFRP composite under C-O-F configuration, although its specific wear rate was lower. In scanning electron microscopy examination, different types of wear mechanisms were revealed in each of the test conducted.

## 1. Introduction

Composite materials have been exploited rapidly by mankind due to their superior characteristics such as higher strength to weight ratio, high impact strength, easily shapeable, and low cost of manufacturing [1]. Today, emerging natural fibre polymer composites have made its way into numerous engineering fields as the world recognizes its biodegradability, eco-friendly and abundantly existent nature. They offer great potential to replace and compete with traditional synthetic fibre, due to their low cost, low density, good relative mechanical properties, good thermal and acoustic insulation properties, reduced respiratory irritation and less damage of tool wear in machining applications [2,3]. One of possible replacement is basalt fibre. Mineral natural fibres are not new, but their suitability as reinforcement in polymer composite is relatively new issue [3,4]. Basalt fibre is an inorganic fibre that has good strength, modulus, better strain to failure than carbon fibre, high operating temperature range, good chemical resistance, excellent heat and sound insulation properties, low water absorption, easily processed, eco-friendly and inexpensive [5,6]. These properties enable basalt fibre to be applied in geopolymeric concrete, pressure pipes, fibrous insulators, protective clothes, naval applications and fire-blocking materials [7,8]. Over the years, Basalt fibres have been hybridized with traditional fibres such as carbon fibre and glass fibre to develop structural materials [4,5,9], and also considered as replacement to traditional fibres by comparing their mechanical properties [3,7,8,10].

Besides mechanical performance, fibre reinforced composite materials are also an important class of tribo-materials. Most industrial, automotive and manufacturing parts such as cams, seals, bearings, shafts, gears, bushes etc. Agrawal et al. [1] and Fazillah et al. are exposed to several types of tribological loading (adhesive, abrasive, erosive etc.) [2]. Therefore, the need to understand their tribological performance is as important as their mechanical performance [11]. Since tribological performance depends on several factors such as operating parameters, characteristic of matrix and fibre, manufacturing process, physical and interfacial properties of fibre, and contact conditions [12], several types of research have been conducted. The different types of sliding conditions such as dry adhesion sliding, abrasion, and erosion, are studied by Zhao et al. [13,14] and Karsli et al. [15]. It can be concluded that wear performance at different sliding conditions does not necessarily relate to one another as different wear mechanisms were involved. As reported by Zhao et al., the performance of Glass fibre and Carbon fibre was best only in adhesive condition, but not in erosive condition [13]. In fact, in erosive condition, all type of reinforcements (Glass, Carbon, Aramid) reduced the wear resistance of polyimide matrix. It was also found that higher temperature environment increases the wear rate at both wear conditions. A similar finding was found by Harsha et al., where elevated temperature reduced the effectiveness of glass fibre reinforcement to improve the wear of PEK working under reciprocating sliding [16]. Conversely, Fazillah et al. found that higher temperature reduced friction coefficient, although it did increase wear rate of oil palm ad kenaf fibre-epoxy composites [2]. Zhao et al. concluded that the type of reinforcement influenced friction coefficient, wear rate and wear mechanism of the polymer matrix, as different type fibre reinforcement performed excellently at dry sliding, three-body abrasive, and reciprocating abrasive wear condition [14]. However, the results of glass fibre and carbon fibre reinforcement displayed more consistent pattern between abrasive and adhesive wear condition [15]. In addition, studies on different wear environments found that water-lubricated [17] and oil-lubricated [1] environments aids in reducing wear rates of FRP composites, compared to dry environment. On the other hand, the effect of operating parameters such as load, velocity, and distance on tribological performance of FRP composite were also studied [10,18,19]. Results found that increase in load will increase friction coefficient and decrease wear rate, while the increase in velocity will increase both friction coefficient and wear rate [10,18]. However, Nasir et al. in their study of unidirectional glass-pultruded-kenaf (UGPK) composite in adhesive sliding revealed a reduction in wear rate as velocity increased, while wear rate reduced before increased again as load increased, indicating a critical value for the composite [19]. Study on a comparison between different types of fibres conducted exhibited that carbon fibre reinforced composite has lower friction and wear rate at different sliding load/velocity compared to the glass fibre [18,20]. Furthermore, Chairman et al. found that the glass fibre shows higher wear rate at different load compared to basalt fibre. The study also showed that wear rate decreased as sliding distance increased [10]. 

Based on the literature review, it is important to have knowledge of the material’s performance under various working conditions, such as applied load, sliding distance, sliding velocity, environment, temperature etc., since tribological properties are not an intrinsic property. There are quite few research studies on the effect of operating conditions and parameters on tribological performance as mentioned above. However, to author best knowledge, studies concerning tribological performance comparison between mineral basalt fibre and synthetic glass fibre at different wear conditions and operating parameters are still limited. Thus, the main aim of present work is a comparative evaluation of friction and wear characteristics of glass fibre and basalt fibre reinforced epoxy composite under different types of wear conditions, motions, parameters, and configurations, to study the effect of reinforcements on tribological performance and wear mechanisms of epoxy-based composites.

## 2. Materials and Methods 

### 2.1. Materials

The epoxy resin used in this study is Miracast 1517 A/B (Density: 1.13 g/cm^3^) supplied by Miracon (M) Sdn. Bhd, Selangor, Malayasia. Miracast 1517A epoxy resin is a low viscosity laminating resin. While Miracast 1517B is an amine-curing agent. The mixing ratio was set at 100:30 as recommended by supplier. Glass fibres (GF) in unidirectional roving form (density: 2.54 g/cm^3^, diameter: 10–17 μm, tensile strength: 3.31 GPa, elastic modulus: 70 GPa) were supplied by Vistec Technology Service (M) Sdn. Bhd, Selangor, Malaysia. Basalt fibres (BF) in unidirectional roving form (density: 2.65 g/cm^3^, diameter: 9–15 μm, tensile strength: 1.96 GPa, tensile modulus: 94.77 GPa, elongation: 1.84%) were supplied by Haining Anjie Composite Material Co.Ltd, Hining, Zhejiang, China.

The composites specimens were produced using conventional filament winding and hand layup method. Frame plates with a dimension of 350 mm × 240 mm were prepared to wind the fibres with a fixed content of 15 vol%. A mixture of epoxy and hardener was distributed evenly onto the frame using a roller. The frame was compressed with 10 N weight to ensure flat surface and thickness before it was left to cure for 24 h at room temperature (RT). The composition and designation of composites specimens prepared in present work are listed in Table 1.

### 2.2. Characterization of the Composites

The density was determined based on Archimedes principle in accordance with ASTM D792. Hardness test was conducted using Instron A654 R Rockwell Hardness (Norwood, MA, USA) tester machine following ASTM D785-08 standard. The calculated density and hardness of composites are presented in Table 2. The morphologies of worn surfaces were examined by Hitachi TM3000 (Illinois, USA) scanning electron microscope (SEM). The specimens were plated with a platinum coating to render electrically conductive using SC7620 QUORUM (Quorum Technologies Ltd., East Sussex, UK) sputter coater machine.

### 2.3. Wear Testing

The friction and wear test was done to evaluate wear rate, the coefficient of friction and wear mechanism of the EP, BFRP and GFRP composites. There were a total of four types of friction and wear test involved that was divided into two stages of tests. The first stage involved dry unidirectional sliding, two-body abrasion, and slurry pot erosion test to investigate wear properties of composites under different wear condition sliding at similar load, speed and distance. The second stage involved dry adhesive sliding under unidirectional and reciprocating motion to investigate wear and friction properties of composites at different type of motions, parameters, and configurations. 

#### 2.3.1. Unidirectional Adhesive Sliding

The dry unidirectional adhesive wear test was performed using TR-20LE model pin-on-disc tribometer (Ducom Instruments, Bohemia, NY, USA) in accordance to ASTM G99-95a standard. The disc shape sample with diameter of 76 mm and thickness of 4 mm was used. While the 10 mm diameter of pin used was GCr15 stainless steel pin with a hardness of HRC 62. In first stage, the test parameters were set at 30 N load, 300 rpm speed and 10 km distance, while in second stage, the test parameters were set at 0.23 MPa·m/s and 0.93 MPa·m/s pressure-velocity (PV) factor and 2 km distance. Before testing, the GCr15 stainless pin was polished with abrasive papers to a surface roughness of about 0.1 μm. The weight of the sample before and after the test was taken by using high precision balance with accuracy of ±0.01 mg. Wear volume (Δ*V*) and Specific wear rate (*Ks*) was calculated using Equations (1) and (2) respectively, as stated in ASTM G99-95a standard. The operating parameters are summarized in Table 3.

(1) $$\Delta V=\frac{\Delta m}{\rho }$$

(2) $$Ks=\frac{\Delta V}{L\times F_{N}}=\frac{\Delta m}{L\times F_{N}\times \rho }$$

where Δ*m* is the mass loss (g) of specimens, *ρ* is the density of sample (g/mm^3^), L is the distance travel (m), and *F_N_* is the normal load (N). 

#### 2.3.2. Two-Body Abrasion Sliding

Two-body abrasion wear test was performed using TR-600 model Abrasion Resistance Tester (Ducom Instruments, Bohemia, NY, USA). The sample was cut into a disc shape of 125 mm diameter and 4 mm thickness. The disc was in contact with two vitrified bonded silicon carbide abrasive wheels, grade 46 (medium coarse). The operating parameters were set at 30 N load, 300 rpm speed and 10 km distance. The weight of the sample before and after the test was taken by using high precision balance with accuracy of ±0.01 mg. Wear volume (Δ*V*) and Specific wear rate (*Ks*) was calculated by using Equations (1) and (2), respectively. The operating parameters are summarized in Table 3.

#### 2.3.3. Slurry Erosion Sliding

The Slurry erosion test was done by using TR-40 model Slurry Erosion Test Rig (Ducom Instruments, USA). The rectangular sample with dimension of 75 mm × 25 mm with a thickness of 4 mm was used. A mixture of medium course (size ranged from 0.2 mm to 0.63 mm) sand and water was used as a slurry item. The surface of specimens was in contact with sand particles that caused erosion on the surface of specimens. The parameters were set at 300 rpm speed and 10 km distance. The weight of the sample before and after the test was taken by using high precision balance with accuracy of ±0.01 mg. Wear volume (Δ*V*) and Specific erosion rate (*Ks*) was calculated using Equations (1) and (3), respectively. The operating parameters are summarized in Table 3.

(3) $$Ks=\frac{\Delta m}{m\times \rho }$$

where Δ*m* is the mass loss of sample (g), m is mass of erodent (g) used, and *ρ* is the density of sample (g/mm^3^).

#### 2.3.4. Reciprocating Adhesive Sliding

The dry reciprocating adhesive wear test was performed using TR-281M8 model. High-frequency reciprocating rig (Ducom Instruments, Bohemia, NY, USA) in accordance with ASTM G133-95 standard. The tribometer operates in a linear repeated back and forth movements creating reciprocating motion. The sample was cut into square shape with a dimension of 15 mm × 15 mm and thickness of 4 mm. The two types of counterface used, which are ball (diameter 6 mm, HRC 60) and cylinder (diameter 6 mm, thickness 9.2 mm, HRC 60) stainless steel, creating ball-on-flat (B-O-F) configuration and cylinder-on-flat (C-O-F) configuration respectively. The operating parameters were set at 70 N load, 500 rpm speed and 120 m distance. Specific wear rate (Ks) of the sample was calculated using Equation (2). The weight of the sample before and after the test was taken using high precision balance with accuracy of ±0.01 mg. The operating parameters are summarized in Table 3.

## 3. Results and Discussion

### 3.1. The Effect of Fixed Load, Speed and Distance under Adhesive, Abrasive and Erosive Wear on BFRP and GFRP Composites

**Figure 1:**
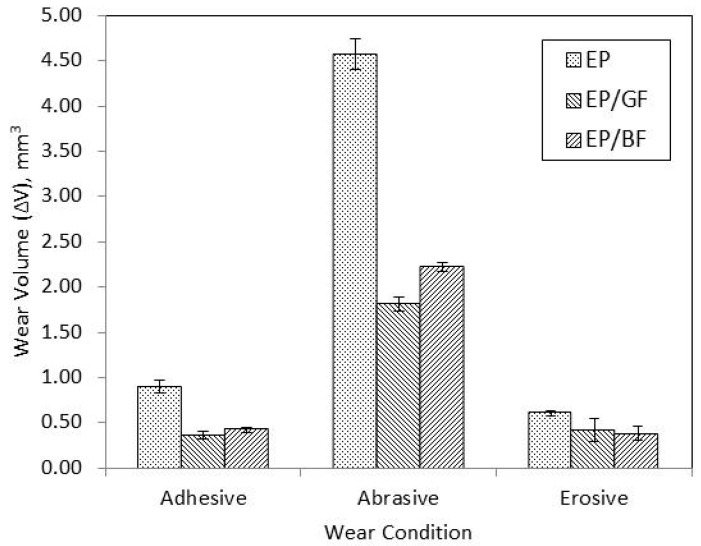
Wear volumes of pure epoxy and its composites at different wear conditions.

Figure 1 shows the comparison of wear volumes (Δ*V*) of EP, EP/GF and EP/BF composite under adhesive, abrasive and erosive wear sliding at 30 N load, 300 rpm speed and 10 km distance. The composites’ performances at long working period under three (3) different wear conditions were observed. As expected, abrasive wear shows highest wear volume amongst the three different wear conditions as the composite was run against very rough vitrified bonded silicon carbide that. Wear volume of adhesive and erosive wear showed almost similar value although one was run in dry condition and the other one was in slurry condition (a mixture of water and sand). This might due to the medium coarse type of sand was used in the slurry mixture. 

Wear properties of the composites were improved when reinforced with glass fibre and basalt fibre in all three different wear conditions. Both reinforcements have shown lower wear volume with improvement up to 60% compared to EP, where the fibres have improved the macroscopic wear resistance of polymers [14]. However, the performance of basalt fibre compared to glass fibre was not consistent in all three wear conditions. For both adhesive and abrasive wear conditions, EP/GF composite showed better wear than EP/BF composite, with differences of 18.33% and 22.75% respectively. This might due to the higher hardness obtained by EP/GF composite compared to EP/BF composite [21] that prevents severe matrix removal from adhesive wear. Besides that, EP/BF composite tend to wear out faster than EP/GF composite might be due to its smaller fibre diameter size, where the basalt fibre’s strength is limited and therefore affecting the fibre-matrix interface strength as well, compared to glass fibre [22]. Only in erosive wear condition did EP/BF composite showed better wear than EP/GF composite, with the improvement of 9.93%. This might attribute to thermal changes that occurred inside the erosive pot. The sand mixture eroding the surface creates higher friction as the distance increases. Therefore, the heating can cause changes to the thermal and mechanical properties of glass fibre reinforcement [13]. However, for all three types of wear conditions, it can be said that BF can potentially replace GF to be used in tribological applications as the differences between them are still comparable.

**Figure 2:**
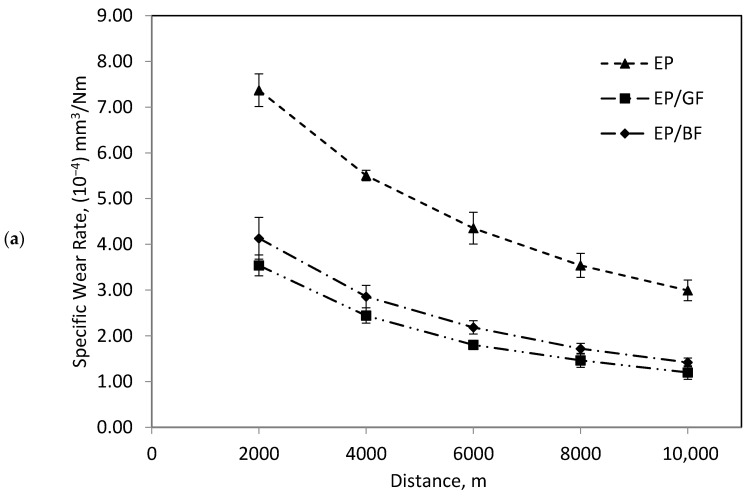

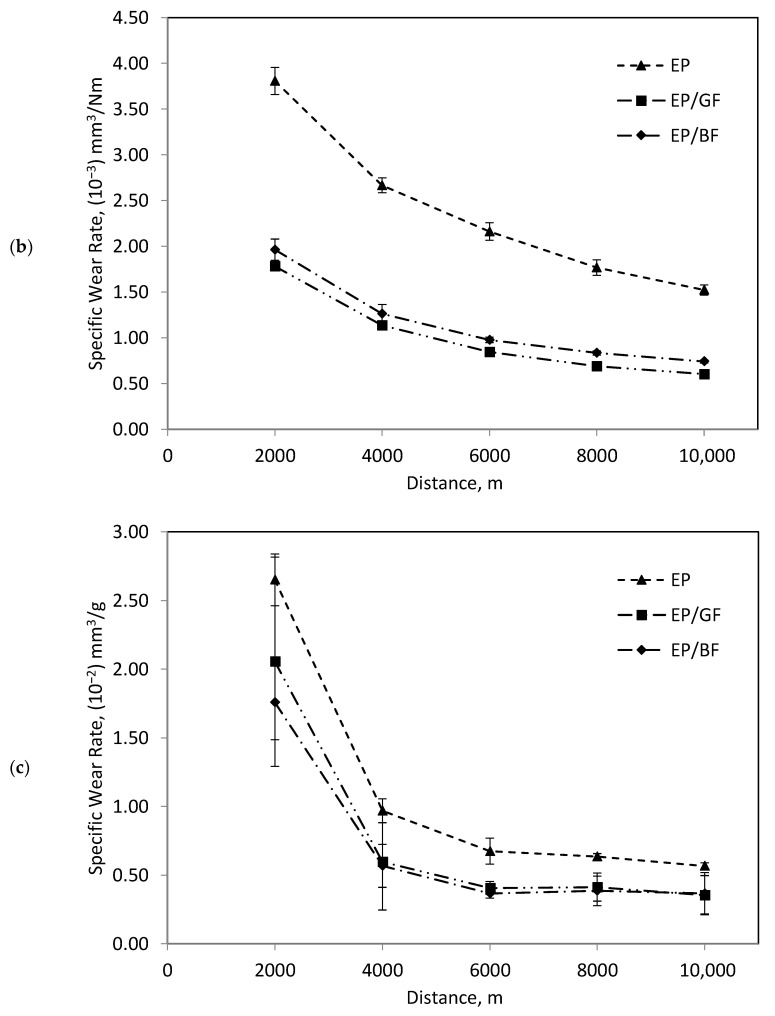
Typical curve of specific wear rate of pure epoxy and its composites under (**a**) adhesive, (**b**) abrasive and (**c**) erosive wear condition.

Figure 2a–c shows a typical trend of wear rate in respect to time for polymeric composite material. The composites experienced a run-in stage for the first 2 km of travel distance where wear rate is high at this stage. As distance increased, wear rate reduced steadily until it attained almost a steady state [23,24]. In Figure 2a, the wear rate eventually decreased due to the formation of transfer film that is believed to develop in running in stage and consequently contributes to the steady wear stage [25]. The transfer film is very common yet very important factor that affects wear rate of adhesive wear for polymeric material. The ability of the polymer to form transfer film and the structure of transfer film itself affects the wear rate of a composite as well as its wear mechanism [13,14,15,26]. In Figure 2b, upon achieving steady-state stage, the decrement of wear rate decreased was due to the fact that the abrasives grit become smoother and less effective than at the beginning of test where the abrasive grit was still fresh [10]. In Figure 2c, as distance increased, the wear rate of reinforced epoxy seemed to be in very close value to each other, where they overlapped. This might be due to the low velocity is chosen, which might be insufficient to observe an obvious difference in erosive performance between composites as the distance increased. 

**Figure 3:**
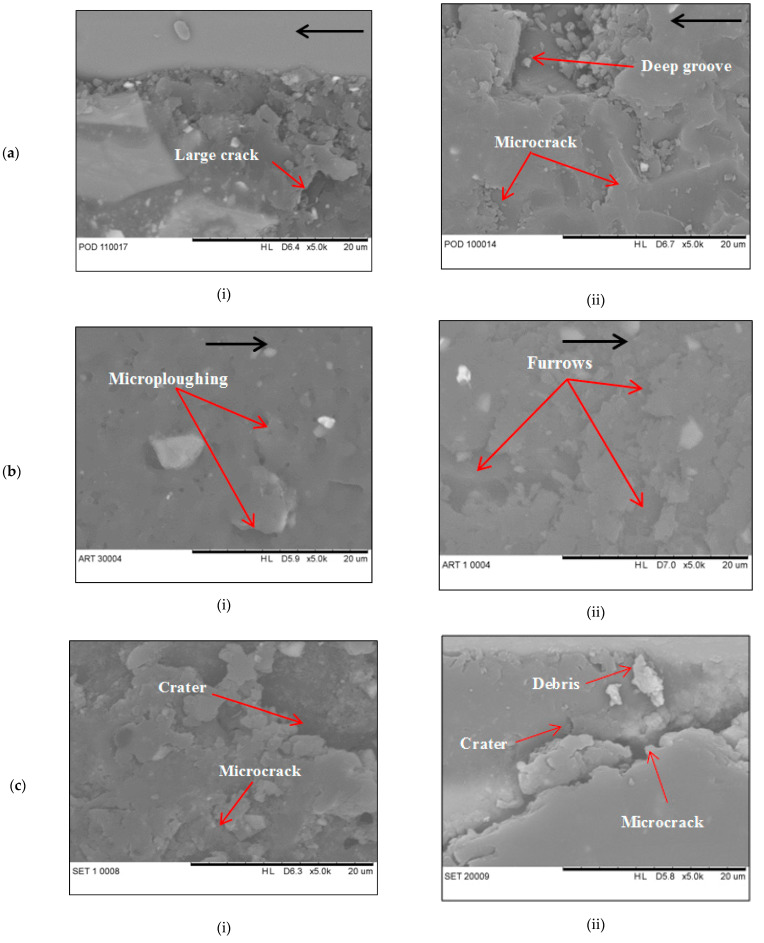
SEM micrograph of worn surface of (i) EP/GF and (ii) EP/BF composites after 10 km travel distance at (**a**) adhesive, (**b**) abrasive and (**c**) erosive wear condition.

Figure 3a,b shows the SEM micrographs of the worn surface of composites after travel for 10 km distance. Under adhesive wear condition, the worn surface of EP/GF composite in Figure 3(ai) shows a roughed and ploughed surface, with the presence of exposed fibre. Microcracks are also visible in the matrix although no cracks are visible on the fibre surface. Some of the fibre exposed is still embedded in the matrix, however, there is also fibre detached from the matrix, showing fibre-matrix debonding. The microcrack in matrix propagates and connects with each other forming a large crack, indicating adhesive wear. For EP/BF composite in Figure 3(aii), the surface is less rough with visible microcrack and exposed fibres. Some of the fibre is still embedded in a matrix, while some are removed, indicating fibre pull-out. There are also deep grooves visible on the matrix, indicating the severe cutting mode of abrasive wear due to the large size of material pull out that leads to its high wear rate value. 

Under abrasive wear condition, EP/GF composite in Figure 3(bi) shows less damage to the matrix. It has less brittle fracture by cutting action. Also, there is very less fibre exposed indicating a strong adhesion between matrix and fibre. There is sign of microploughing at matrix showing small brittle fracture by cutting action by the abrasives. EP/BF composite in Figure 3(bii) shows more damage to the matrix with some fibre visible and exposed. The fibres are still intact in a matrix with no fibre pull out indicating good adhesion between matrix and fibre. There is sign of furrows in matrix indicating brittle fracture by cutting action of the abrasives. In erosive wear, EP/GF composite in Figure 3(ci) shows severe matrix erosion with microcracks formation visible. The fibre also exposed with some fibre showing sign of breakage and fibre-matrix debonding. However, in EP/BF composite in Figure 3(cii) shows less severe erosion, only small portion of fibre were exposed. In both figures, the microcracks are visible at most areas, where they propagate towards each other leaving small groove, pit and craters. They were chipped off of the matrix surrounding the fibre, leaving debris behind. The crater formed mostly resulted from cutting mechanism from the impact of erosion sand. Mild abrasive wear is the predominant wear mechanism for both composites.

### 3.2. The Effect of Low and High PV Factor under Unidirectional Sliding Motion

**Figure 4:**
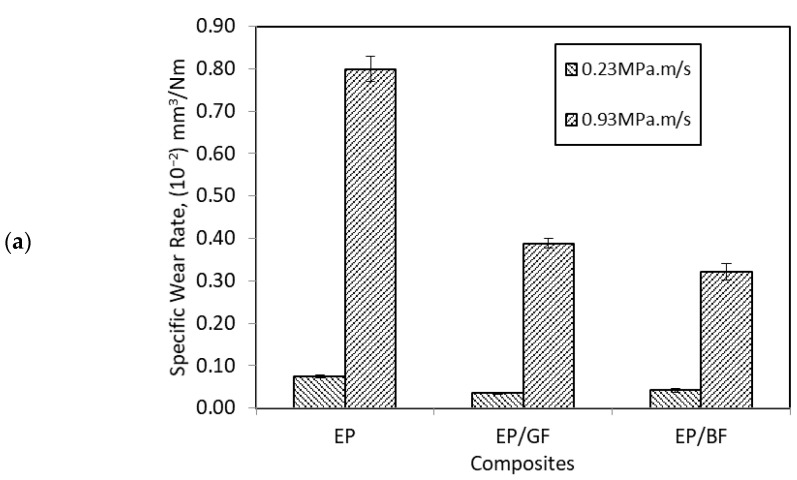

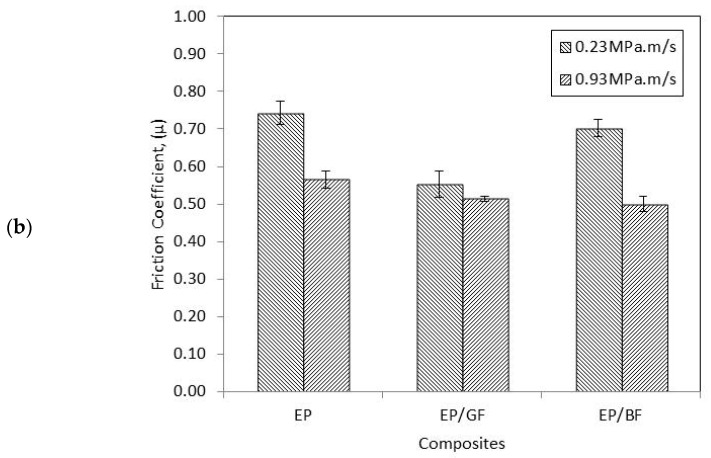
(**a**) Specific wear rate and (**b**) friction coefficient of pure epoxy and its composites under low and high PV factor of unidirectional sliding.

Figure 4 shows the specific wear rate and friction coefficient of EP, EP/GF, and EP/BF composites sliding in a unidirectional direction at low and high PV factor. In Figure 4a the wear rate for FRP composites shows a very distinct value at different load and velocity. At higher parameter, wear rate increased by one order of magnitude for all composites. The range of wear rate for high PV are between 0.04–0.07 × 10^−2^ mm^3^/Nm, while for low PV the wear rate ranges from 0.30–0.80 × 10^−2^ mm^3^/Nm. This trend is consistent with the general relationship of wear rate with load and velocity, where, as load and/or velocity increased, wear rate should increase as well [27]. The effect of thermal softening of the epoxy polymer at higher load and velocity due to increased frictional heat might lead to higher wear rate as the thicker soft surface will be detached from the composite [26]. As shown in Figure 4a, wear properties of the epoxy polymer were improved up to 60% when reinforced with fibre, at both high and low PV factor. Fibre reinforcement carries most load and protect polymer from severe wear [13], either in low or high PV. However, between the FRP composites, wear rate trend did not show similar trait at low and high PV. At higher PV, EP/BF composite showed better wear rate than EP/GF composite with 17.49% improvement, different than at low PV where EP/GF composite then showed better wear rate with 16.72% difference with EP/BF composite. It can be said that effect of basalt fibre reinforcement was more prominent at higher PV.

In Figure 4b, the μ of all FRP composites shows a slightly comparable value when sliding at low and high PV factor, unlike their wear rate value. Overall, it can be seen that μ at high PV has lower and more consistent value than μ at low PV. When sliding at high PV, there was not enough time to produce more adhesive points due to the decrease in surface contact time. The lack of frictional force component by adhesion has led to easier and faster formation of integrated transfer film that eventually reduces the surface softening by frictional heat [28]. Besides that, the high pressure also forces big particle shaped debris to be crushed and sheared into thinner flakes that act as lubricant, therefore, contribute to lower two-body abrasive wear [21]. On the other hand, the figure shows that the μ of epoxy polymer was enhanced when reinforced with fibre at both low and high PV, up to 25% improvement. At low PV, EP/GF composite shows lower μ value than EP/BF composite, with a difference of 27.35%. However, at high PV, EP/BF composite shows lower μ value than EP/GF composite, with the small improvement of 2.95%. Due to the high load and velocity, the broken GF might become third body abrasive that scratches the steel counterface since it is brittle and sharp in nature, therefore increasing its μ since its wear rate was also high [13].

**Figure 5:**
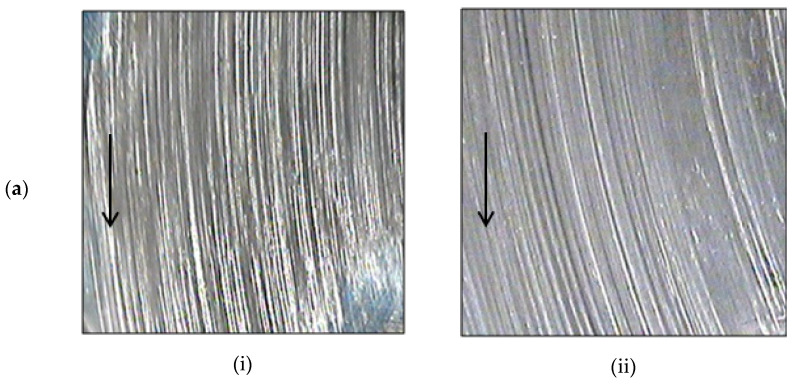

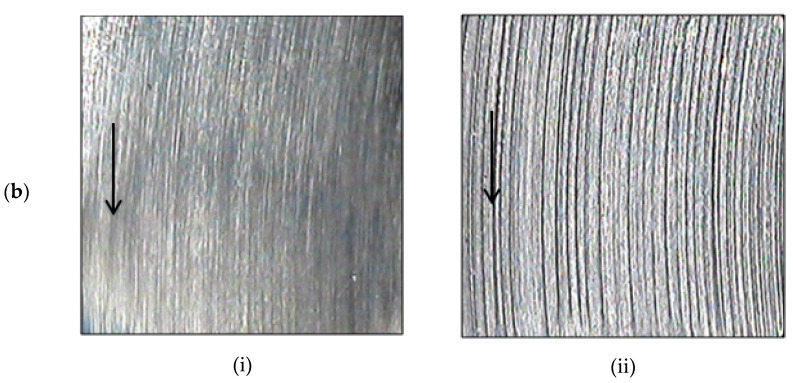
Optical micrograph of transfer film on steel counterface that slide against (**a**) EP/GF and (**b**) EP/BF composites at (i) 0. 23 MPa.m/s and (ii) 0. 93 MPa.m/s PV factor.

Transfer film greatly influences wear behaviour of adhesive wear sliding where the transfer film reduce adhesion between polymer composite and counterface creating a polymer-on-polymer contact instead of polymer-on-metal contact [25]. Figure 5 shows the optical micrograph of transfer film formed on metal counterface (pin) that slide against EP/GF and EP/BF composites at low and high PV factor. From general observation, each counterface has a layer of transfer film that covers the surface in contact with finish undergoing the sliding test. The presence of continuous transfer film has aided in reducing direct contact with asperities of the steel counterface [14], thus reducing its ploughing action [26]. From the micrographs, the condition of transfer film can be observed more clearly. Transfer film at high PV (Figure 5(aii,bii) was more uniform and integrated, that resulted in lower μ compared to transfer film at low PV. Transfer film for low PV (Figure 5(ai,bi)) were less integrated and quite lumpy. These signs resulted in higher μ for the composites.

**Figure 6:**
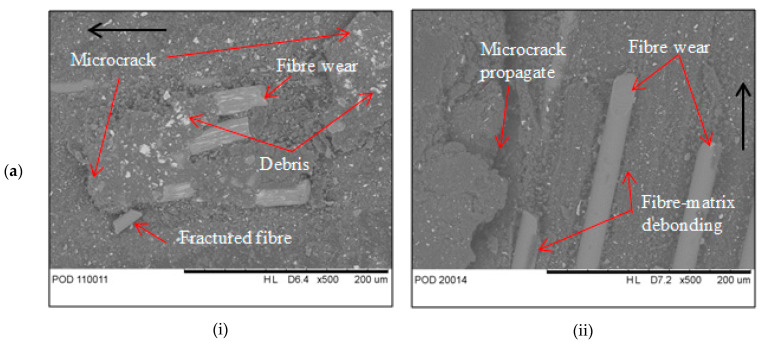

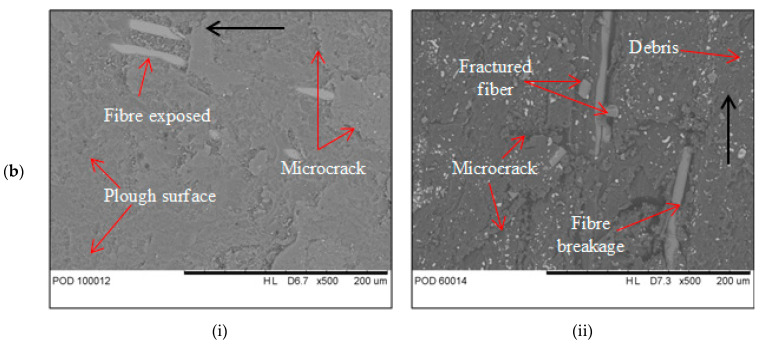
SEM micrograph of worn surface of (**a**) EP/GF and (**b**) EP/BF composites at (i) 0. 23 MPa.m/s and (ii) 0. 93 MPa.m/s PV factor.

Figure 6 shows SEM micrograph of worn surface of EP/GF and EP/BF composites at low and high PV factors. At low (Figure 6(ai)) composite showed severe matrix wear with some fibre exposed and wear off. The microcracks formed on matrix are visible especially at interfacial region. There was also small sized fibre deposited on the worn surface indicating that at some region, the sublayer of matrix supported the fibre has completely worn off, exposing the fibre which the fibre wear off and fractured after repeated shear loading. At high PV (Figure 6(aii)), the matrix wear was more severe with more fibre exposed. The microcrack at matrix surface has propagated and most has peeled off due to surface fatigue which usually occurred at high temperature [29]. The fibre has debonded and wears off since no matrix are protecting it from shear loading. At low PVEP/BF (Figure 6(bi)) composite showed less matrix damage with less fibre exposure. The surface was rough and ploughed where adhesive wear is dominating and ploughing. At high PV, EP/BF (Figure 6(bii)) composite showed more severe matrix damage with mild fibre exposure and fibre breakage. The surface was rough with brittle fragments and multiple microcracks visible at various regions, indicating wear by brittle fracture and abrasive wear. Some fibres are exposed indicating gradual wear of matrix, where the fibres support most load after the matrix has removed off, with some fractured and detached off. The detached fibre has introduced back to the surface, creating three body abrasives.

### 3.3. The Effect of Ball and Cylinder Counterfaces under Reciprocating Sliding Motion

**Figure 7:**
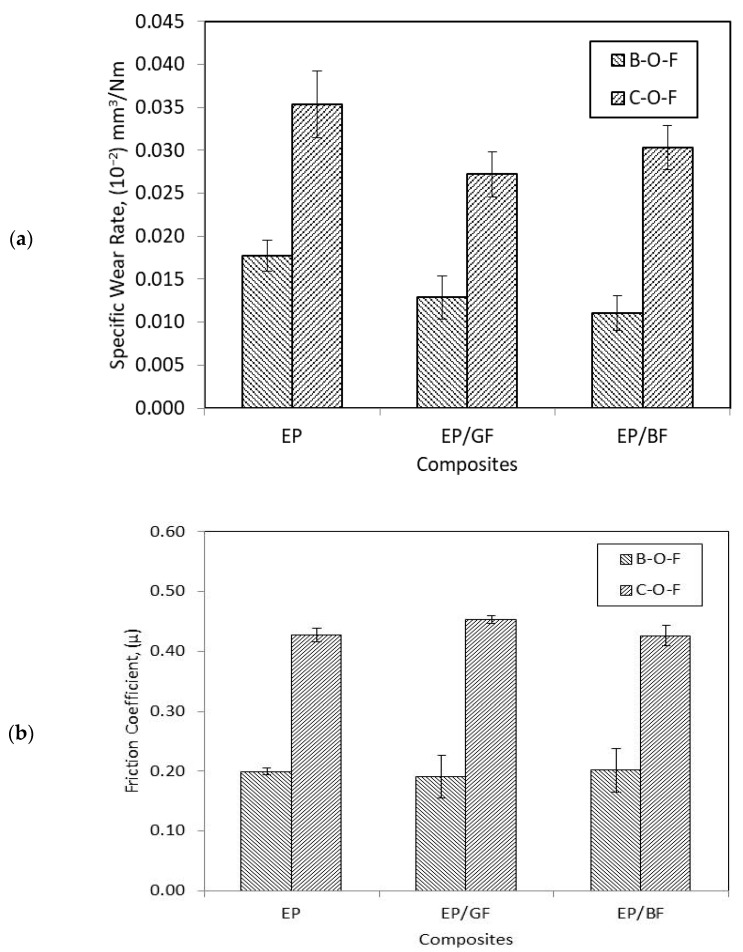
(**a**) Specific wear rate and (**b**) friction coefficient of pure epoxy and its composites under B-O-F and C-O-F configuration of reciprocating sliding.

Figure 7 shows the specific wear rate and friction coefficient of EP, EP/GF, and EP/BF composites sliding in reciprocating motion against ball (ball-on-flat) and cylinder (cylinder-on-flat) counterface. In general, wear rate of Ball-on-flat (B-O-F) configuration have a lower value than wear rate of Cylinder-on-flat (C-O-F) configuration. This is due to the higher pressure of B-O-F configuration since it has a smaller surface contact area which resulted in a lower value of wear rate for the composites [29,30]. The smaller range value of wear rate for B-O-F also showed a more stable wear mechanism. As shown in Figure 7a, the wear rate of epoxy polymer was improved up to 37% with the reinforcement of fibres, at both configurations. For FRP composites, the wear rate shows the different trend between the two configurations. At B-O-F configuration, EP/GF composites have slightly higher value than EP/BF composites, while at C-O-F configuration, the trend is reversed. EP/BF composite shows 13.95% improvement, and 12.22% deterioration compared to EP/GF composite for B-O-F and C-O-F configurations respectively. This might be due to different wear mechanism involved in sliding of ball and cylinder steel counterface on the FRP composites, which affect the wear resistance of the composites.

In Figure 7b, the μ for B-O-F configuration was almost two times (2×) lower than C-O-F configuration. The reason behind smaller μ value was due to the fact that B-O-F configuration has smaller contact area compared to C-O-F configuration, as mentioned before. The smaller contact area makes the pressure at the worn surface higher, thus lowering the result of μ and also wear rate [29,30]. Besides that, the μ value is different in set/range from one configuration to another due to the nature of μ value which is not an intrinsic property, but it strongly depends on the tribological system the material involved with [16]. Between epoxy and its composites, the μ exhibited slight improvement of 4.4% under B-O-F configuration. There was also slight deterioration in μ, might be due to the brittle and abrasive nature of the fibres [13]. The broken fibres may scratch the surface and therefore increased the μ. In B-O-F configuration, EP/GF showed lower μ than EP/BF composite, with 5.72% difference. Meanwhile, in C-O-F configuration, the μ has shown otherwise, were EP/BF composite has improved the μ by 5.96% compared to EP/GF composite. Although the difference is not large, the reason behind this might due to different wear mechanism that occurred at two different contact areas (ball and cylinder steel counterface) on the FRP composites, which affect the μ of the composites.

**Figure 8:**
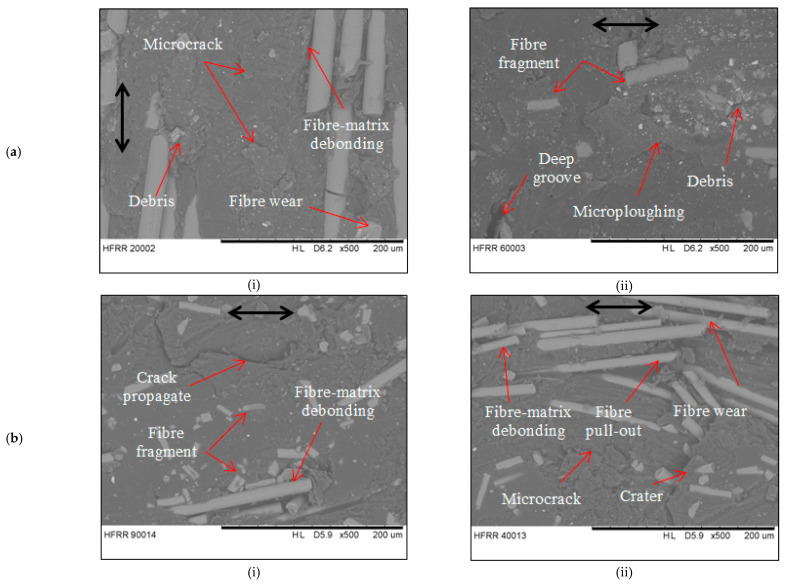
SEM micrograph of worn surface of (**a**) EP/GF and (**b**) EP/BF composites at (i) B-O-F and (ii) C-O-F sliding configuration.

Figure 8 shows the SEM micrograph of the worn surface of EP/GF and EP/BF composites at B-O-F and C-O-F configurations. Based on the micrographs, overall observation can be made on the surface removal and fibres exposure that they are severely affected. One important observation to take note from these micrographs is the condition of the exposed fibres. The fibres originally are all oriented in one direction, which is parallel to the sliding direction of the counterface. At the end of the test, however, the fibres seem to be displaced from their original orientation and re-arranged themselves randomly without specific direction. This might be due to the type of motion of the test; i.e., back and forth at high-frequency reciprocating motion. The reciprocating-type motion has believed to make the removed material to be trapped in between the surface and the counterface (back-transferred) especially for B-O-F configuration where the pressure exerted at the dented wear track is deeper and smaller in size. Therefore, the fibres that have been fractured and pulled outbreaks even more into smaller pieces and embedded again into the matrix since it is soft due to high-temperature rise, resulted in scattered fibres as seen at the worn surface.

Figure 8 displays matrix wear, fibre fracture and interfacial debonding visible at various places. The matrix removal for EP/GF composite in B-O-F configuration Figure 8(ai) are severe with a lot of fibre exposure. The matrix has microcracks and signs of surface fatigue all over the region, due to ploughing action from adhesive wear. Some of the fibres have signs of interfacial debonding between its surrounding matrix. The fibres surface also has a sign of wear, indicating that the fibre has high adhesion strength with the matrix [15]. For C-O-F configuration, in Figure 8(aii), the matrix has deep grooves at certain regions indicating microblogging wear, caused by adhesion. The fibres exposed are very less, but some fragments of fibres can be seen embedded in the matrix. There are also sign of fibre wear at the fibre surface. For EP/BF composite in B-O-F configuration, (Figure 8(bi)), the matrix surface is smooth at the certain region with scattered fibre exposure and fibre fragments at the various region. Cracks propagation can be seen at the top region, indicating surface fatigue, with the sign of fibre debonding and fibre pull out nearby, showing that the shear loading is bigger than the interfacial strength between fibre and matrix. In C-O-F configuration, in Figure 8(bii), the matrix shows a rough surface with a lot of fibre exposed and fibre fragments. Microcracks are seen propagating to form small craters that will be removed after repeated loading. There is fibre wear visible at end of exposed fibre together with fibre-matrix debonding. The backscattered fibres and wear debris embedded in certain region are due to the reciprocating motion of the counterface. Main wear mechanism has changed from adhesive wear to abrasive wear.

## 4. Conclusions

Friction and wear characteristics of epoxy composites reinforced with glass fibre (EP/GF) and basalt fibre (EP/BF) were evaluated under different wear conditions, motions, parameters and configurations. The following conclusions can be drawn:

- Incorporating fibres into epoxy polymer matrix affects the tribological properties of epoxy. Under different wear conditions namely adhesive, abrasive and erosive wear, working for long time period showed improved wear properties up to 60% when epoxy was reinforced with fibres. Under unidirectional sliding, FRP composite improved wear rate and friction coefficient of epoxy polymer up to 60% and 25% respectively. And lastly, under reciprocating sliding, wear rate and friction coefficient was improved up to 37% and 4% respectively, compared to epoxy polymer.
- Low friction coefficient recorded during sliding does not always reflect a low wear rate of the composite. Friction coefficient was high during low operating parameters of unidirectional sliding, although its wear rate was low.
- EP/BF composite did not perform well when working for a long time period as it has lower wear properties than EP/GF composite in adhesive and abrasive wear conditions. Only in erosive wear did EP/BF composite has better wear properties than EP/GF composite, improved by slightly 9.93%. All the composites experience run-in stage at first 2 km distance travel which is typical for polymeric material before reaching steady-state stage.
- In comparison to EP/GF composite, under unidirectional sliding, EP/BF composite has shown better wear rate and friction coefficient only at high operating parameters with improvement up to 17.49% and 2.95% respectively, where it can be said that basalt fibre work more effective under high operating parameters. The transfer film formed was more uniform and integrated resulted in lower friction coefficient. Wear mechanism involved at low parameter was adhesive wear and ploughing, while at high parameter, wear mechanism involved brittle fracture and abrasive wear.
- During reciprocating sliding, EP/BF composite showed an inconsistent pattern of improvement between wear rate and friction coefficient, compared to EP/GF composite. Wear rate was improved at B-O-F configuration by 13.95%, however, its friction coefficient did not follow suit. Only at C-O-F configuration did friction coefficient was lower with 5.96% improvement compared to EP/GF composite. Wear mechanism involved in B-O-F and C-O-F configuration was surface fatigue and abrasive wear respectively.
- Type of reinforcement and tribological system influences the wear rate, friction coefficient and wear mechanism serving as indicators for selection of composites for specific applications.

## Figures and Tables

**Table 1:** Composition and designation of the composites.

No.	Composites	Designation
1	Epoxy	EP
2	Epoxy + 15 vol% Glass Fibre	EP/GF
3	Epoxy + 15 vol% Basalt Fibre	EP/BF

**Table 2:** Density and hardness of the composites.

No.	Composites	Density (g/cm^3^)	Hardness (HRR)
1	EP	1.157 ± 0.003	122.53
2	EP/GF	1.375 ± 0.014	122.55
3	EP/BF	1.324 ± 0.009	119.50

**Table 3:** Summary of operating parameters for friction and wear tests.

Stage	Test	Parameters	Distance	Counterface	Environment
1	Unidirectional Adhesive sliding	30 N, 300 rpm	10 km	Stainless Steel Pin	Dry, RT
	Abrasive Sliding	30 N, 300 rpm	10 km	Vitrified bonded Silicon Carbide	Dry, RT
	Erosive Sliding	300 rpm	10 km	Mixture of sand and water	Wet slurry, RT
2	Unidirectional Adhesive sliding	0.23 MPa.m/s 0.93 MPa·m/s	2 km	Stainless Steel Pin	Dry, RT
	Reciprocating Adhesive sliding	70 N, 500 rpm	120 m	Stainless steel ballStainless steel cylinder	Dry, RT

## Data Availability

Not Applicable.
